# Clinical Characteristics and Prognosis of Acute Pulmonary Embolism with Hemoptysis in Autoimmune Disease Patients

**DOI:** 10.7150/ijms.94052

**Published:** 2024-05-19

**Authors:** Yiyao Li, Jianian Yang, Peijun Xue, Ting Zhang, Xuefeng Sun, Min Peng, Juhong Shi

**Affiliations:** 1 Department of Pulmonary and Critical Care Medicine, State Key Laboratory of Complex Severe and Rare Diseases, Peking Union Medical College Hospital, Beijing, China.; 2 Chinese Academy of Medical Sciences, Peking Union Medical College, Beijing, China.

**Keywords:** Pulmonary Embolism, Prognosis, Anticoagulants, Treatment

## Abstract

**Background:** Hemoptysis is prevalent in acute pulmonary embolism (PE) and significantly influences clinical decision-making. Despite the increasing reports of PE in patients with autoimmune diseases, limited studies have investigated the association between acute PE with hemoptysis and autoimmune disease.

**Methods:** The retrospective study aimed to investigate patients with autoimmune disease who presented with acute PE and hemoptysis at Peking Union Medical College Hospital (PUMCH) between January 2012 and October 2020. A comparative analysis was conducted between patients with and without hemoptysis, as well as between those with autoimmune diseases and those without. Clinical characteristics, PE severity stratification, the amount of hemoptysis, initial anticoagulation management, and prognosis were analyzed descriptively.

**Results:** The study analyzed 896 patients diagnosed with acute PE, of whom 105 (11.7%) presented with hemoptysis. Hemoptysis in PE patients was frequently associated with autoimmune diseases (39%, 41/105), a younger patient population (42.0 vs. 52.7 years old, *P* =0.002), and a higher prevalence of low-risk PE (53.7 vs. 28.1, *P*=0.008) compared with non-autoimmune disease patients. Multivariate logistic analysis showed PE patients with primary or metastatic lung cancer, chest pain, age < 48 years old, chronic heart failure, autoimmune disease, pulmonary infection and male were more likely to develop hemoptysis. Patients were grouped based on maximum daily sputum blood volume and PE risk stratification. Most patients (73.2%) received therapeutic-dose anticoagulation. Poor prognosis is observed in patients with moderate to massive hemoptysis and intermediate-high-risk or high-risk PE.

**Conclusions:** Hemoptysis is a relatively common manifestation in patients with PE, and its presence during the diagnostic workup of acute PE necessitates careful analysis of underlying comorbidities. In cases where hemoptysis occurs in individuals with autoimmune diseases in the context of PE, proactive management strategies targeting the primary disease are crucial. Therapeutic decisions should consider both PE severity stratification and the volume of hemoptysis.

## Background

Pulmonary embolism (PE) is the world's third leading cause of cardiovascular death 1. Acute PE can present variably, with an incidence of hemoptysis ranges from 2.6% to 7.6% 2-4. The etiology of hemoptysis in patients with acute PE may be attributed to the PE itself, such as embolism-associated pulmonary infarction. It is also crucial to consider the potential underlying diseases that may contribute to this manifestation.

The presence of hemoptysis in patients diagnosed with acute PE poses a challenging diagnostic and therapeutic dilemma, and current guidelines do not offer specific management strategies 5-7. Hemoptysis caused solely by acute PE is usually of mild to moderate volume, and it does not affect anticoagulation strategies 8, 9. Conversely, when hemoptysis is unrelated to PE, the initiation of anticoagulant therapy requires careful consideration of the balance between thrombosis severity and bleeding risk, with priority given to treating coexisting conditions.

PE is a common complication of autoimmune disease, which presents a therapeutic challenge in when it occurs in patients with hemoptysis. Given the heterogeneous etiology of hemoptysis in patients with infections and tumors, differential clinical analysis is imperative. Available evidence indicates that inflammation plays an important role in increasing the risk of venous thromboembolism (VTE) in patients with autoimmune disease by promoting hypercoagulability and endothelial damage 10, 11. In certain systemic vasculitis and connective tissue diseases, the diseases can directly affect the pulmonary vasculature, resulting in extreme high-risk PE and massive hemoptysis in certain conditions 12-14. Managing both the underlying disease and the concurrent thrombotic event presents clinical challenges.

Early identification of patients with autoimmune disease and a concurrent PE, as well as aggressive management of the underlying disease and proper balancing of the risk of thrombosis and hemoptysis, are crucial to improving the prognosis of these patients. However, only a limited number of studies have specifically investigated the relationship between acute PE with hemoptysis and autoimmune disease.

This study aimed to investigate the clinical characteristics, treatment approaches, and outcomes of autoimmune patients with PE and hemoptysis. Furthermore, PE severity stratification and volume classification of hemoptysis were utilized to evaluate the optimal treatment options and prognosis for different levels of severity.

## Materials and Methods

### Study design

This is a retrospective, observational study conducted at a single-center. We enrolled consecutive inpatients aged 18 years or older who diagnosed with acute PE at Peking Union Medical College Hospital (PUMCH) between January 1, 2012, to October 22, 2020. The inclusion criteria for diagnosis and severity stratification followed the guidelines provided by the European Society of Cardiology (ESC) 15. Confirmation of PE required computed tomographic pulmonary angiography (CTPA), enhanced computed tomography of the chest, and scintigraphic ventilation-perfusion (V/Q) scan revealing a high probability of PE. Transthoracic echocardiography was performed to assess right ventricular (RV) function. Exclusion criteria included chronic PE and non-thromboembolism such as bone-cement embolism and lipiodol embolism were excluded. Patients with insufficient baseline clinical data for analysis were also excluded.

### Data collection

The patient data included in the analysis, such as age, gender, symptoms, signs, comorbidities, as well as laboratory and radiological findings, are results obtained at the time of PE diagnosis. The therapeutic measurement including anticoagulation strategies refer to the initial strategies upon hospital admission, and the in-hospital outcomes pertain to the observing results during the hospital stay. Pulmonary embolism severity index (PESI) 5 and simplified pulmonary embolism severity index (sPESI) 5 were calculated for all patients. Cardiac biomarkers used in the risk assessment of PE include cardiac troponin I (cTnI), and N terminal pro-B-type natriuretic peptide (NT-proBNP). Patients presenting with cardiac arrest, obstructive shock, or persistent hypotension were defined as high-risk PE. Patients who had positive sPESI score together with either right ventricular dysfunction (by echocardiography or CTPA) or elevated cardiac biomarker levels in the circulation (elevated cTnI or natriuretic peptide concentrations in plasma) were classified as intermediate-risk patients. PE patients with 0 score of sPESI were classified as low-risk. Standard therapeutic-dose anticoagulation was defined as weight-adjusted or full-treatment doses of low-molecular-weight heparin (LMWH) (eg, dalteparin 100 units/kg every 12 h or enoxaparin 1 mg/kg every 12 h). Intermediate-dose anticoagulation was defined as generally 0.5 mg/kg of enoxaparin twice daily or 1 mg/kg of enoxaparin once daily, or an equivalent 16.

### Variable definition and clinical endpoint

Based on the classification methods reported in previous studies 9, 17 and the actual distribution of hemoptysis volume in this study, we divided the patients into three groups by the maximum daily blood volume in sputum: (i) Mild hemoptysis: ≤20 mL/24h, (ii) Moderate hemoptysis: 20<volume≤100ml, (iii) Massive hemoptysis: >100mL/24h. Patients were classified into four groups based on the volume of hemoptysis and PE severity stratification (**Fig. 4**). Group A is defined as patients with mild hemoptysis and low-risk or intermediate-low- risk PE. Group B is defined as patients with moderate to massive hemoptysis and low- risk or intermediate-low-risk PE. Group C is defined as patients with mild hemoptysis and intermediate-high-risk or high-risk PE. Group D is defined as patients with moderate to massive hemoptysis and intermediate-high-risk or high-risk PE. The primary endpoint was all-cause death and major bleeding event during hospitalization. Major bleeding was defined according to the definition proposed by the International Society on Thrombosis and Hemostasis (ISTH) 18. All patients were followed up after discharge until April 21, 2021, with confirmation of outcome events. The follow-up data was ascertained by interviewing patients, families, or their physicians using the telephone. This study was approved by the Institutional Review Board of the Peking Union Medical College Hospital (PUMCH) (Ethical review number: B164) according to the Declaration of Helsinki. Informed consent was signed by the participants or their authorized family members.

### Statistical analysis

Continuous and integral variables were presented as the mean value and standard deviation (SD) for normally distributed variables, and the median and quartile were used for abnormal distributed variables. Categorical variables were expressed as their counts and proportions. Patients with and without autoimmune disease were compared by two-sided independent Student's t-test for normally distributed variables, Mann-Whitney U test for variables obeyed abnormal distribution, and chi-square test or Fisher's exact test for categorical data. A logistic regression model was used to identify the risk factors for hemoptysis. Continuous variables were transformed into binary variables for logistic regression analysis, and the cut-off value was determined by receiver operating characteristic (ROC) curve analysis. Next, the covariates of multivariate analysis were selected from the univariate analysis with statistical significance (*P* < 0.05). We selected variables with clinical significance and those with a *P*-value < 0.05 in the univariate regression analysis to enter into the multivariate regression analysis. Variables exhibiting multicollinearity were excluded, specifically through the diagnosis using tolerance and variance inflation factor (VIF). Variables with a tolerance less than 0.1 and a VIF greater than 5 were excluded. Odds ratio (OR) and the corresponding 95% confidence interval (CI) were reported. All tests were two-sided, and *P* < 0.05 was considered to indicate statistical significance. Statistical analysis was conducted using R version 4.2.3.

## Results

### Baseline characteristics of patients with PE and hemoptysis

**Figure 1:**
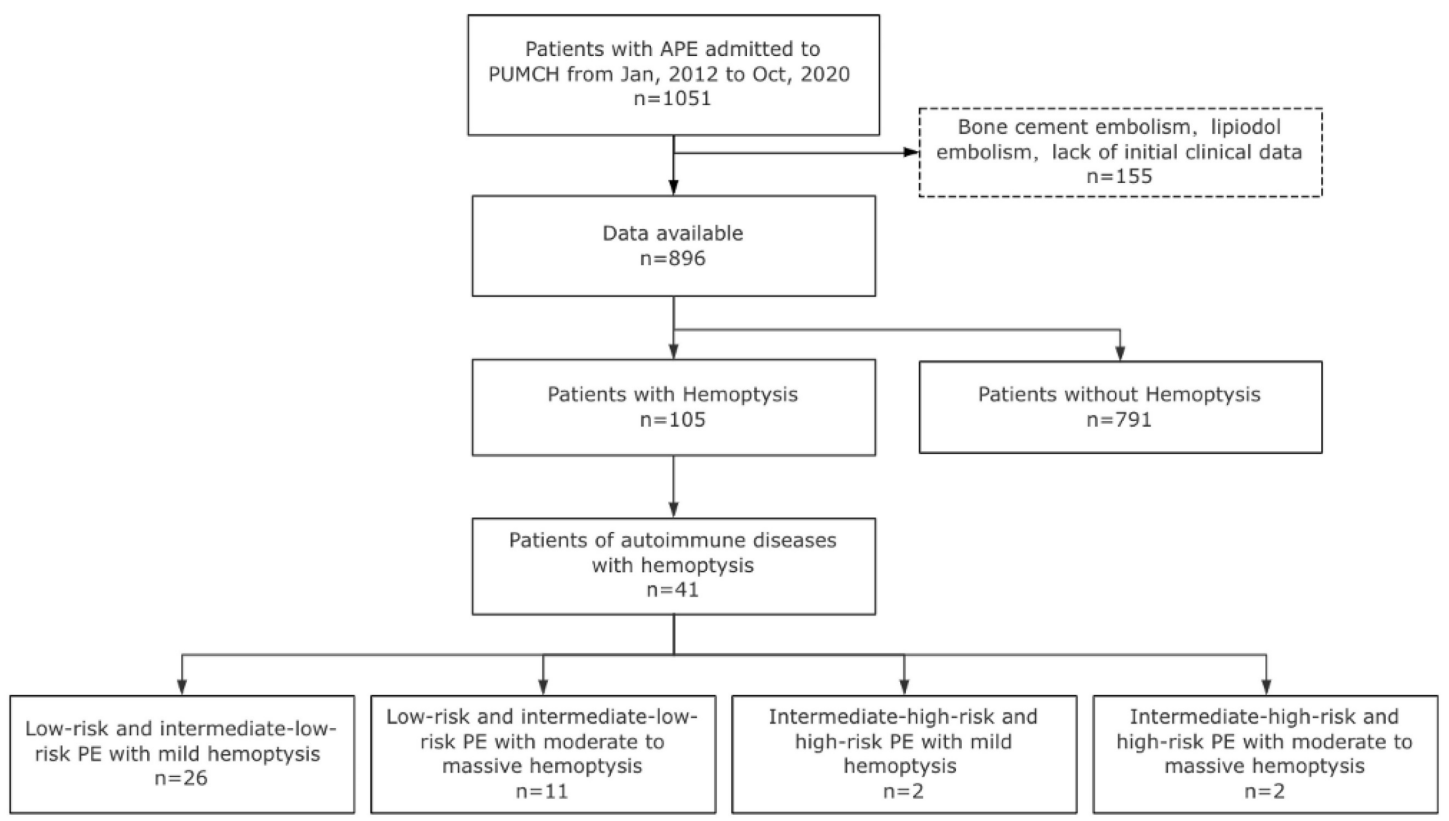
The flowchart of the study.

**Figure 2:**
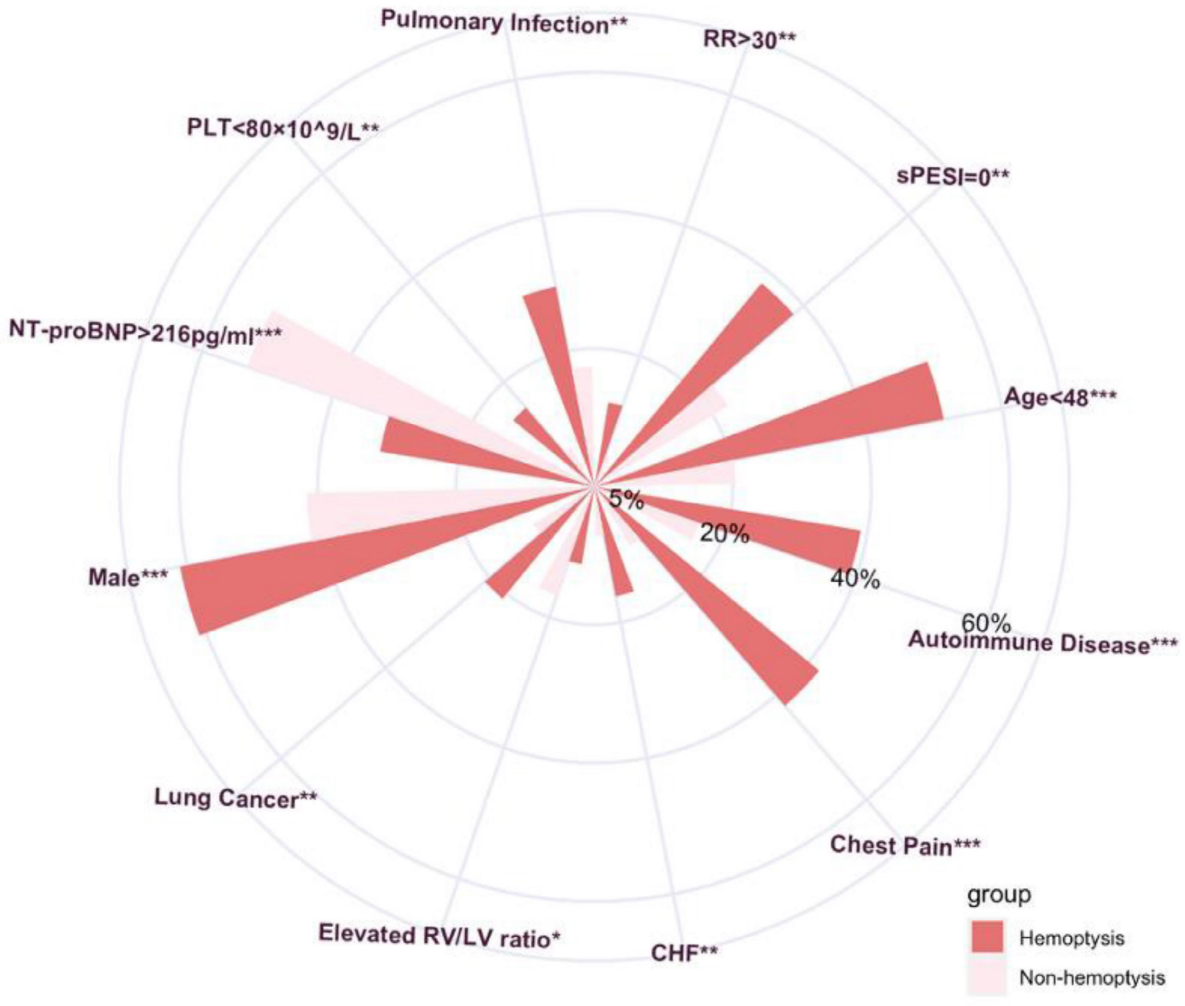
Clinical characteristics with statistically significant differences of PE patients with hemoptysis versus patients without hemoptysis. Continuous variables were transformed into binary variables, and the cut-off value was determined by ROC curve analysis. RR, respiratory rate, breaths per minute; sPESI, simplified pulmonary embolism severity index; CHF, Chronic Heart Failure; RV, right ventricle; LV, left ventricle; Elevated RV/LV ratio, dilated RV with basal RV/LV ratio >1.0. NT-proBNP, N-terminal pro-B-type natriuretic peptide; PLT, platelet. *** P < 0.001.** P < 0.01.* P < 0.05.

Between January 2012 and October 2020, a total of 896 adult patients with acute PE were included in the study (**Fig. 1**). Among them, 105 patients presented with hemoptysis at the diagnosis time of PE diagnosis, resulting in an incidence rate of 11.7%. The clinical characteristics of patients with PE and hemoptysis were compared to those without hemoptysis, as shown in **Table S1**. Variables with statistically significant differences are illustrated in **Fig. 2**. Results showed that patients with hemoptysis were more likely to have autoimmune diseases (39.0% vs. 16.1%, *P* < 0.001), particularly systemic vasculitis and antiphospholipid syndrome (APS). Additionally, other autoimmune diseases such as systemic lupus erythematosus (SLE), Sjögren's syndrome (SS), rheumatoid arthritis (RA), undifferentiated connective tissue disease (UCTD), and idiopathic inflammatory myopathies (IIM) were also observed within the studied population. Furthermore, a high prevalence of pulmonary infection (29.5%), primary or metastatic lung cancer (21.0%), and chronic heart failure (16.2%) was observed in the hemoptysis group (**Table S1**).

### Clinical factors related with hemoptysis

**Figure 3:**
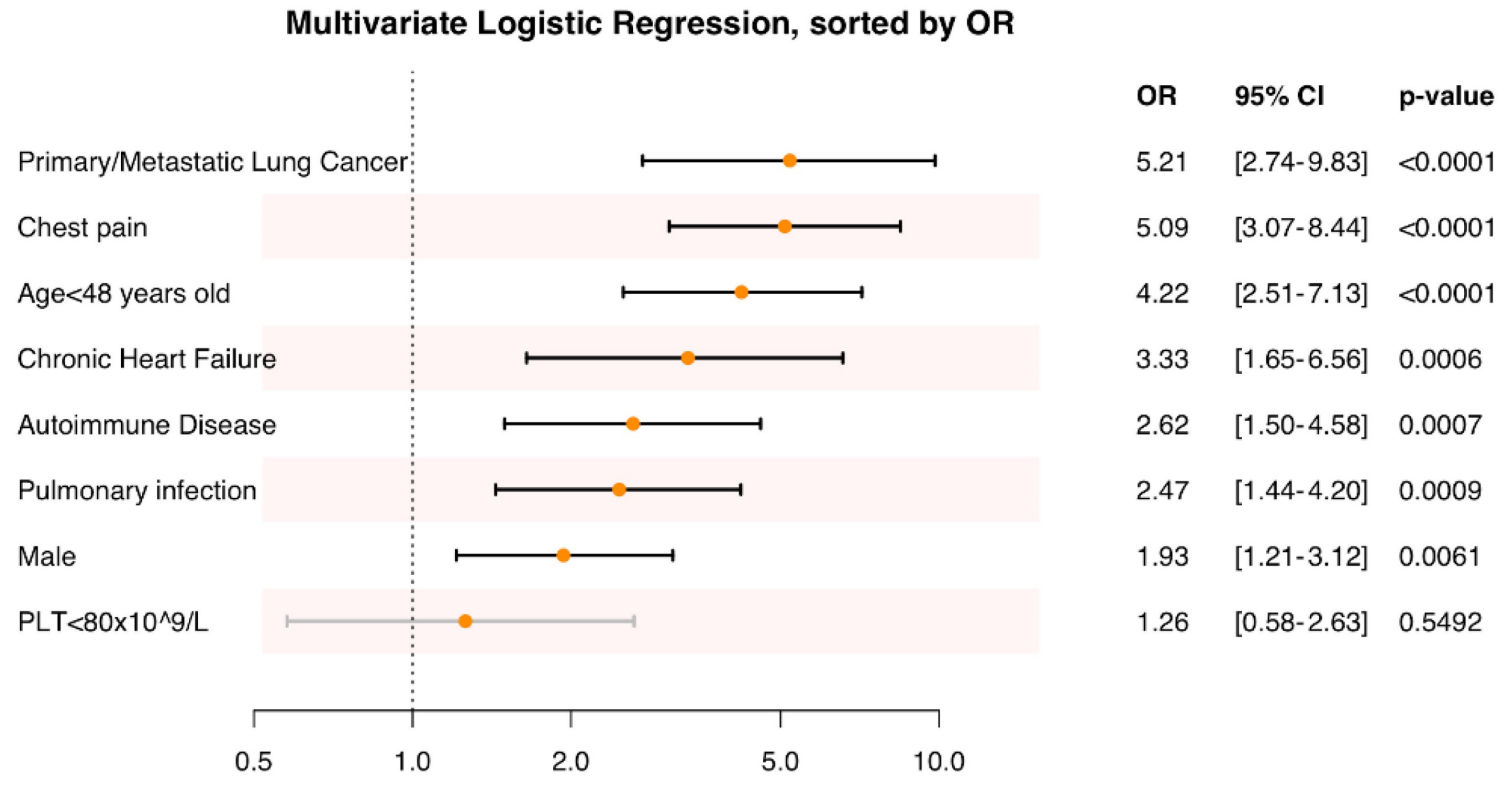
Multivariate analysis of clinical factors associated with hemoptysis in PE patients.

Multivariate logistic analysis was performed to further confirm the independent risk factors for hemoptysis in PE patients. The variables included in the multivariate analysis were male gender, age < 48 years old, PLT<80×10^9^/L, chest pain, and comorbid diseases (all *P* values<0.05, **Table S2**). The result is presented in **Fig. 3** along with the corresponding OR (95% CI). The increased risk of hemoptysis ranged from an OR of 1.93 (95% CI: 1.21-3.12) for males to an OR of 5.21 (95% CI: 2.74-9.83) for primary or metastatic lung cancer. Other independent risk factors associated with hemoptysis in PE are chest pain (OR 5.09, 95% CI 3.07-8.44, *P* <0.001), age < 48 years old (OR 4.22, 95% CI 2.51-7.13, *P* < 0.001), chronic heart failure (OR 3.33, 95% CI 1.65-6.56, P < 0.001), autoimmune disease (OR 2.62, 95% CI 1.50-4.58, *P* < 0.001), and pulmonary infection (OR 2.47, 95% CI 1.44-4.20, *P* <0.001).

### Comparison of clinical characteristics between PE with hemoptysis in patients with and without autoimmune disease

**Figure 4:**
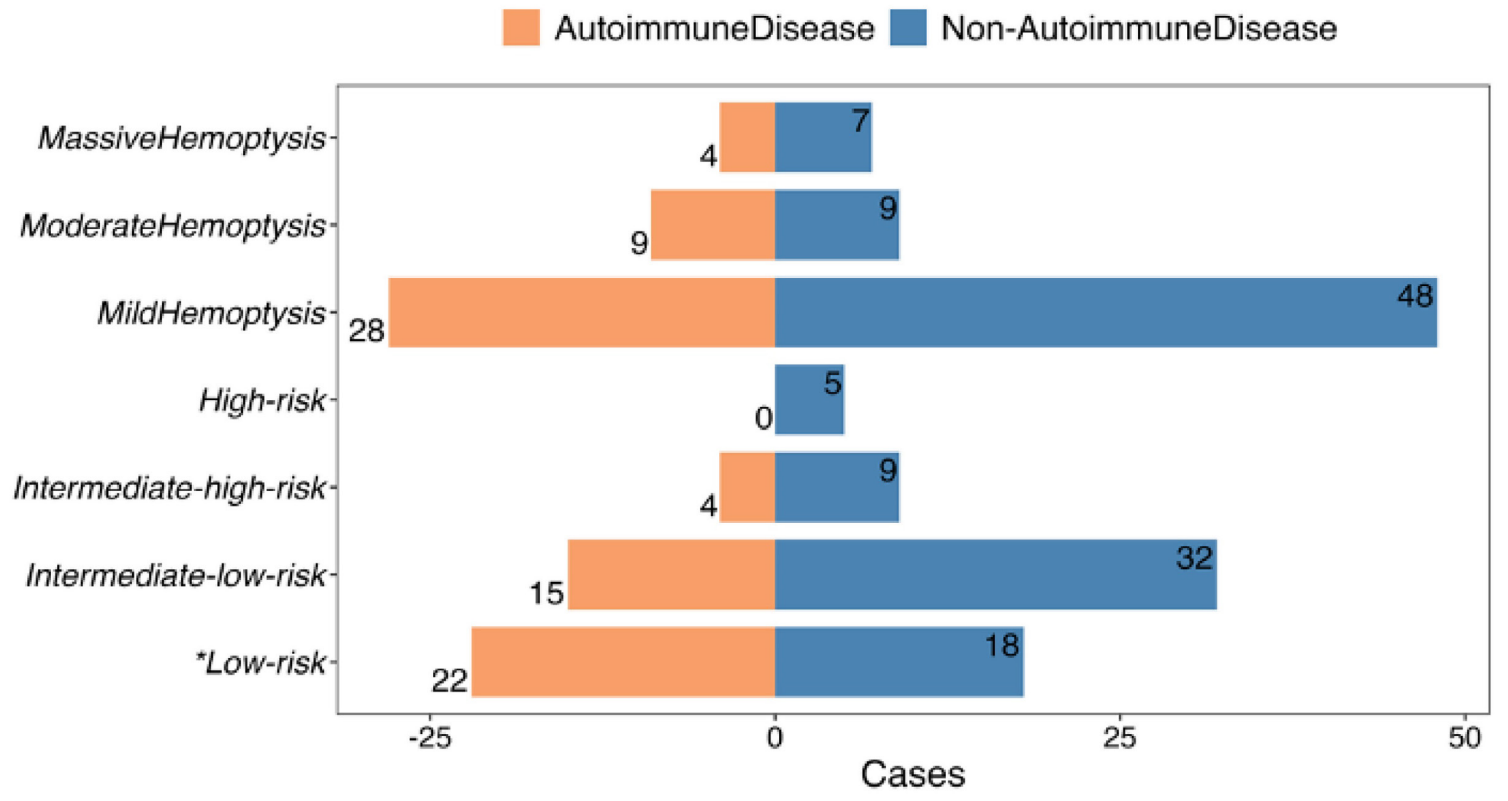
PE severity and amount of hemoptysis of autoimmune disease patients versus non-autoimmune disease patients. * The difference is statistically significant.

Having established autoimmune diseases as a risk factor for PE with hemoptysis, characteristics within this specific subgroup were further investigated. Patients with autoimmune disease and PE presenting with hemoptysis were found to be younger (42.0±17.7 vs. 52.7±17.6 years, *P* = 0.002) and had a lower frequency of dyspnea (39.0% vs. 65.6%, *P* = 0.013), compared to those without autoimmune disease (**Table 1**). No significant differences were observed in other vital signs or thrombus locations in CTPA and echocardiography between the two groups (**Table 1**). A higher proportion of patients with a “0” sPESI score (53.7 vs. 28.1%, *P* = 0.015) (**Table 1**) and low-risk PE (53.7 vs. 28.1%, *P* = 0.015) (**Fig. 4**) were observed in the autoimmune disease group. The severity of hemoptysis in this patient group was classified as mild (28 cases, 68.3%), moderate (9 cases, 22.0%), and massive (4 cases, 9.7%) (**Fig. 4**).

### In-hospital Outcomes in four groups stratified by PE Risk and hemoptysis volume in autoimmune disease patients

Among the cohort of 105 patients presenting with hemoptysis, a total of 15 patients (14.3%) experienced mortality during their hospitalization, including 3 patients with prolonged hospital stays exceeding 30 days. Two patients experienced major bleeding following anticoagulation therapy, both of whom were diagnosed with lung cancer and presented with concomitant massive hemoptysis. The comparison of short-term mortality rates between the hemoptysis and non-hemoptysis groups did not reveal any statistically significant differences (**Table S3**).

**Figure 5:**
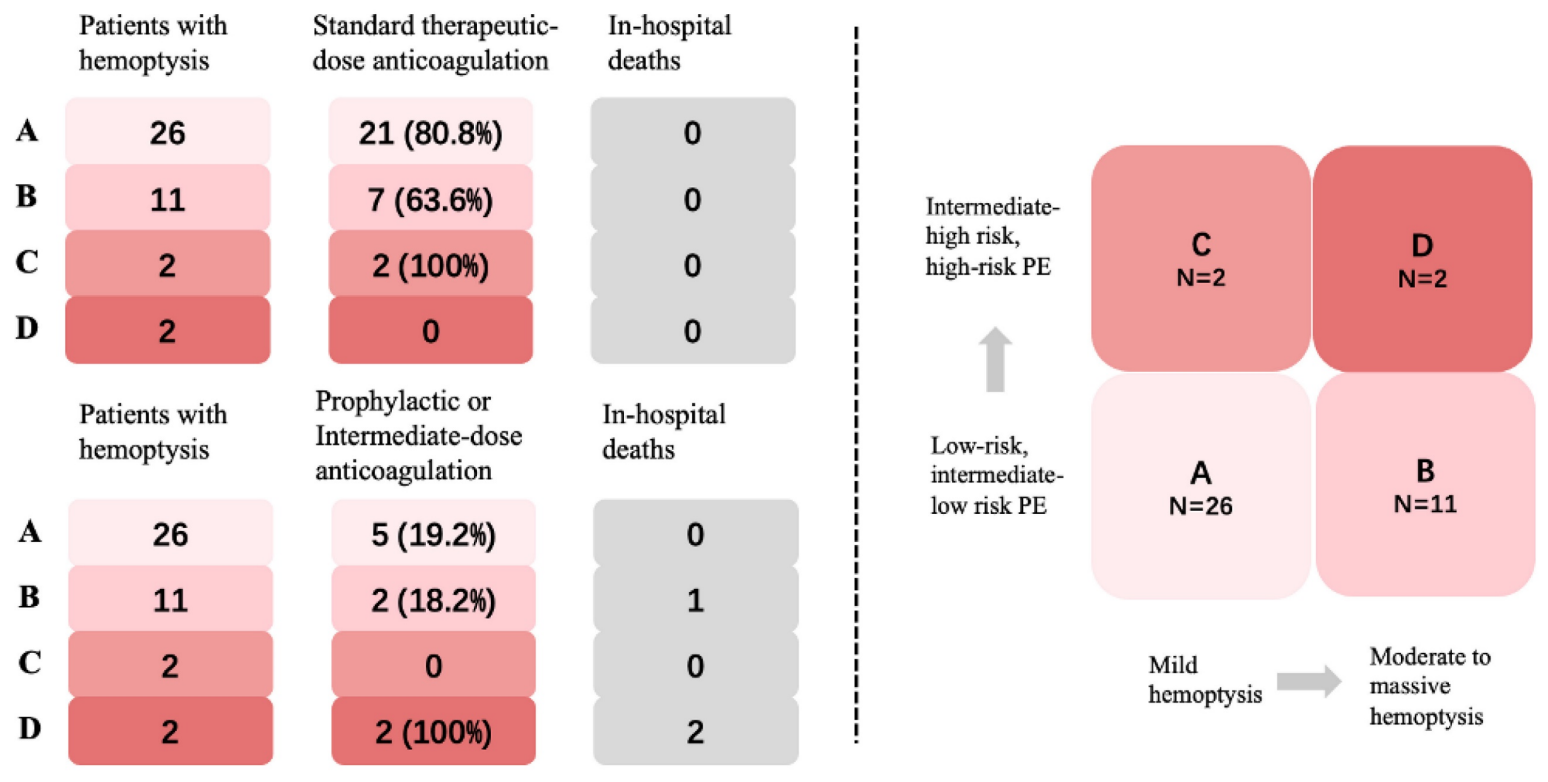
Classification, anticoagulation managements, and outcomes of the four groups.

Patients with autoimmune diseases who experienced both hemoptysis and PE demonstrated a lower 30-day all-cause mortality rate when compared to patients without autoimmune diseases and none of them experienced major bleeding after anticoagulation. Notably, no significant disparity was observed in the in-hospital mortality rate between these two groups (**Table 1**). In general, 73.2% of the patients received standard therapeutic-dose anticoagulation, and there were no in-hospital deaths observed. Among the remaining 21.9% of patients who received intermediate-dose anticoagulation, three cases resulted in mortality due to comorbidities, without any exacerbation of hemoptysis or occurrence of major bleeding. Additionally, 4.9% of the patients did not receive anticoagulation (**Fig. 5**).

In Group A, 80.8% (21/26) of patients received therapeutic-dose anticoagulation (**Fig. 5**). Another 5 (19.2%) had intermediate-dose anticoagulation due to hemoptysis. In Group B, 63.6% (7/11) of patients underwent standard therapeutic-dose anticoagulation with no reported cases of hemoptysis exacerbation. A reduction in anticoagulation was seen in 36.4% (4/11) of patients, including 2 patients with Behçet disease (BD) who did not receive anticoagulation. One patient died from vasculitis with severe infection, but there were no cases of progressive PE. All patients in Group C received therapeutic-dose anticoagulation without further hemoptysis. Two patients in Group D received intermediate-dose anticoagulation, both died due to severe comorbidities. The first patient had multi-organ involvement of systemic lupus erythematosus (SLE) and died of gastrointestinal bleeding, despite treatment with glucocorticoid shock, cyclophosphamide, and plasmapheresis. The second patient had rheumatoid arthritis-related interstitial lung disease (RA-ILD) combined with severe infection, resulting in respiratory failure and death.

## Discussion

This study, we analyzed the clinical characteristics, anticoagulation managements, and prognostic outcomes of patients with autoimmune disease and PE presenting with hemoptysis at PUMCH over an eight-year period from 2012 to 2020. Results showed that the incidence of hemoptysis was 11.7%. Multivariate logistic analysis showed PE patients with primary or metastatic lung cancer, chronic heart failure, autoimmune disease, and pulmonary infection were more likely to develop hemoptysis. 39% of patients with hemoptysis had autoimmune diseases, making this the largest category of comorbidities among patients with hemoptysis. Consequently, we conducted a detailed analysis of the characteristics of this subgroup of patients. These patients were younger and had a higher prevalence of low-risk PE. All patients received appropriate treatment for their primary diseases, with 73.2% received therapeutic-dose anticoagulation. Poor prognosis is observed in patients with moderate to massive hemoptysis and intermediate-high-risk or high- risk PE.

For comorbidities that predispose to hemoptysis, patients with autoimmune diseases represent a distinct subgroup, as the causes of their hemoptysis differ from those with other comorbid conditions. Hemoptysis can occur when lung malignancies directly invade blood vessels, or when pulmonary lesions caused by tissue necrosis or inflammation lead to rupture of bronchial arteries 19, 20. Patients with chronic heart failure are more prone to hemoptysis due to prolonged pulmonary congestion and edema. Autoimmune disease patients often experience a heightened inflammatory state, and when combined with systemic vasculitis or other connective tissue diseases involving pulmonary vasculitis, hemoptysis may result from direct damage to the vessel wall due to infiltration of inflammatory cells, leading to loss of vascular integrity and subsequent bleeding 21, 22. Moreover, a hypercoagulable state has been linked to the hyperinflammatory state during disease activity in autoimmune diseases 23, 24, with the development of VTE predicting disease activity 25, 26. This has led to the observation that individuals with autoimmune diseases are at a high risk for both thrombosis and hemoptysis, making the coexistence of these two conditions not uncommon.

We propose a prioritized approach for managing such cases in autoimmune disease. Firstly, aggressive treatment of the primary disease is essential. In this study, vasculitis was most prevalent among autoimmune diseases, with 37.5% had ANCA-associated vasculitis (AAV), 25% had Behçet's disease, 12.5% had Takayasu arteritis, and 25% had unclassified vasculitis. Treatment decisions for vasculitis must be made based on the specific type of vasculitis and the type of blood vessels affected by the disease. In AAV, besides presenting with occasional mild hemoptysis, diffuse alveolar hemorrhage (DAH) can manifest as massive hemoptysis or remain subtle, initially appearing as mild hemoptysis. Hemoptysis in patients with APS possibly associated with the high incidence of PLT reduction 27. In BD, up to 40% of patients may present with vascular lesions, with pulmonary aneurysms and hemoptysis being the most common symptoms 28-30. In Takayasu arteritis, thickening and stenosis of the pulmonary artery wall may lead to hemoptysis in approximately half of the patients with pulmonary artery involvement 31, 32. Other connective tissue diseases (CTD) may also exhibit pulmonary involvement, leading to interstitial changes, small vessel vasculitis, and occasionally mild hemoptysis.

Among the patients with low- or intermediate-low-risk PE with moderate to massive hemoptysis (group B), which accounted for 26.8% (11/41) of the total, 7 (63.6%) underwent therapeutic-dose anticoagulation, 2 (18.2%) had anticoagulation at the intermediate dose, and 2 cases of BD combined with pulmonary aneurysm were not anticoagulated. Notably, one patient with APS and DAH, presenting with moderate to massive hemoptysis, was treated with therapeutic-dose anticoagulation. Glucocorticoids and immunosuppressant agents were prescribed for this patient, and he received plasmapheresis. With standard-dose anticoagulation, the patient did not experience further exacerbation of DAH and was discharged with improvement. Tseng *et al.* 33 conducted a retrospective study of five patients with vasculitis who developed both PE and DAH. One patient had anticoagulation discontinued due to DAH and underwent inferior vena cava filter placement after treatment with glucocorticoids, cyclophosphamide, and plasmapheresis. However, one month later, the patient experienced recurrent PE. The remaining four patients continued anticoagulation therapy without exacerbation of hemoptysis or recurrent thrombosis during follow-up. For patients with similar conditions, reducing anticoagulation dosage to alleviate hemoptysis may have a risk of recurrent PE, as suggested by limited case experience. Therefore, clinicians should exercise caution when considering decreasing anticoagulation in such patients 33, 34. Within this category, there were two patients diagnosed with BD and PE, who presented with hemoptysis greater than 100 ml. In these cases, anticoagulation was not applied, and the primary disease was managed with the addition of glucocorticoids and immunosuppressive therapy. BD patients often demonstrate pulmonary artery involvement with both pulmonary aneurysm and pulmonary embolism, with a combination of PE observed in 23.5% (8/34 cases) of patients with BD who have pulmonary aneurysm formation 35. Thrombosis in patients with BD is primarily the result of systemic inflammation of the vessel wall, rather than abnormalities in coagulation. As such, treatment of vascular inflammation in BD is prioritized over anticoagulation, and it is currently believed that most patients' thrombosis can be improved without anticoagulation when the primary disease is controlled 36-38.

Secondly, balancing the volume of hemoptysis with the severity of PE allows for the appropriate management of the most critical aspect. In group C, we observed that a small proportion (4.8%, 2/41 cases) of patients with intermediate-to-high or high-risk PE with mild hemoptysis did not experience any further worsening of hemoptysis after receiving aggressive treatment of the primary disease and standard therapeutic-dose anticoagulation. Of the patients with low- risk or intermediate-low-risk PE with mild hemoptysis (group A), 63.4% (26/41 cases) had anticoagulation at a therapeutic dose, and 80.8% (21/26 cases) of these patients did not experience any adverse events leading to death. A small number of patients (19.2%, 5/26 cases) received intermediate-dose anticoagulation, with 2 cases reduced due to combined thrombocytopenia and 3 cases reduced due to concerns about hemoptysis. Notably, the therapeutic-dose anticoagulation did not exacerbate hemoptysis in patients with PE, and patients with mild hemoptysis are recommended to receive aggressive treatment for their underlying disease.

Of course, there are also extremely critical cases. A minority of patients (4.9%, 2/41) with autoimmune disease were identified as intermediate-to-high risk or high-risk PE with moderate to massive hemoptysis (group D). Despite the relatively low incidence, the management of such cases poses significant clinical challenges. One patient with multisystem involvement of SLE, with an sPESI score of 3, developed DAH. Although the patient received glucocorticoid pulse therapy, cyclophosphamide, and plasmapheresis for SLE, along with intermediate-dose anticoagulation, she ultimately succumbed to gastrointestinal bleeding. Another patient had RA-ILD with severe infection, sPESI score 4, and moderate to massive hemoptysis of fresh blood in sputum, received intermediate-dose anticoagulation and eventually died due to progressive respiratory failure. The cause of death in both patients was likely attributed to the severity of their comorbidities. Given the small number of cases, the optimal approach to managing severe PE and massive hemoptysis in this population remains inconclusive.

Several limitations need to be taken into consideration. As a retrospective study, there might be potential inaccuracies in estimating the total daily amount of hemoptysis based on medical records. Despite being a single-center study, as the largest center for the diagnosis and treatment of rare and complex diseases in China, our patient population and sample size still provide insight into the national status of difficult and rare diseases. Further investigation through a prospective, multicenter study is required to better understand the clinical features and treatment strategies in this patient population.

## Conclusions

The incidence of hemoptysis in hospitalized patients with acute PE was 11.7%. PE patients with primary or metastatic lung cancer, chronic heart failure, autoimmune disease, and pulmonary infection were more likely to develop hemoptysis. Among patients with hemoptysis, 39% had an autoimmune disease; these patients were younger and more likely to have low-risk PE compared to those with other underlying diseases. Treatment should prioritize appropriate management of the autoimmune disease and stratification of treatment based on the severity of PE and the amount of hemoptysis is also recommended.

## Supplementary Material

Supplementary tables.

## Figures and Tables

**Table 1:** Clinical characteristics of acute PE with hemoptysis in autoimmune disease patients versus non-autoimmune disease patients

	Total		Patients with autoimmune disease presenting with hemoptysis (n=41/105)		Patients without autoimmune disease presenting with hemoptysis (n=64/105)		*P*-value	
**Male, n (%)**	64 (61.0)		24 (58.5)		40 (62.5)		0.846	
**Age, year, mean ± SD**	48.9±18.5		42.0±17.7		52.7±17.6		0.002*	
**Symptoms, n (%)**								
Chest pain	44 (41.9)		22 (53.7)		22 (34.4)		0.079	
Dyspnea	58 (55.2)		16 (39.0)		42 (65.6)		0.013*	
Syncope	5 (4.8)		1 (2.4)		4 (6.3)		0.646	
**Signs, n (%)**								
Pulse ≥ 110 beats/min	16 (15.2)		5 (12.2)		11 (17.2)		0.677	
RR > 30 breath/min	13 (12.4)		4 (9.8)		9 (14.1)		0.562	
SBP < 100 mmHg	13 (12.4)		4 (9.8)		9 (14.1)		0.562	
SpO_2_<90%	26 (24.8)		8 (19.5)		18 (28.1)		0.444	
**Laboratory findings**								
WBC (× 10^9^/L), median (IQR)	8.41 (5.79,12.28)		8.27 (5.32,11.27)		8.11(5.73,12.28)		0.974	
HGB (g/L), mean±SD	124.85±22.419		122±21.7		120±24.0		0.390	
PLT (× 10^9^/L), mean±SD	204±107.8		178±109.8		220±103.7		0.050	
D-dimer (mg/L), median (IQR)	4.31 (2.07,11.81)		3.09 (1.57,9.32)		4.51 (2.42,13.32)		0.127	
cTnI ≥ 0.056ug/L, n (%)	22 (21.0)		7 (17.1)		14 (21.9)		0.788	
NT-proBNP (pg/ml), median (IQR)	180 (125,1285)		125 (125,688)		213 (125,2835)		0.089	
Cr (umol/L), median (IQR)	70 (58,84)		71 (55,83)		70 (62,87)		0.932	
**Comorbid Diseases, n (%)**								
Coronary atherosclerotic heart disease	10 (9.5)		2 (4.9)		8 (12.5)		0.301	
Chronic Heart failure	17 (16.2)		7 (17.1)		10 (15.6)		>0.99	
Pulmonary infection	29 (27.6)		9 (22.0)		20 (31.3)		0.415	
Primary/Metastatic lung cancer	22 (21.0)		1 (2.4)		21 (32.8)		<0.001*	
Cancer	29 (27.6)		2 (4.9)		27 (42.2)		<0.001*	
**Echocardiography, n (%)**								
Elevated RV/LV ratio	12 (11.4)		4 (9.8)		8 (12.5)		0.531	
RV free wall hypokinesis	8 (7.6)		2 (4.9)		6 (9.4)		0.275	
sPAP≥50mmHg	14 (13.3)		5 (12.2)		9 (14.1)		0.129	
**CTPA, n (%)**								
Central emboli	11 (10.5)		5 (12.2)		6 (9.4)		0.900	
**sPESI, n (%)**								
0		40 (38.1)		22 (53.7)		18 (28.1)		0.015*
1		32 (30.5)		10 (24.4)		22 (34.4)		0.386
2		20 (19.0)		6 (14.6)		14 (21.9)		0.505
3		11 (10.5)		2 (4.9)		9 (14.1)		0.195
4		2 (1.9)		1 (2.4)		1 (1.6)		>0.99
In-hospital deaths, n (%)		15 (14.3)		3 (7.3)		12 (18.8)		0.153
								

SD, standard deviation; RR, respiratory rate; SBP, systolic blood pressure; WBC, white blood cell; COPD, chronic obstructive pulmonary disease; DPLD, diffuse parenchymal lung disease; IQR, interquartile range; HGB, hemoglobin; PLT, platelet; cTnI, cardiac troponin I; NT-proBNP, N-terminal pro-B-type natriuretic peptide; RV, right ventricle; LV, left ventricle; sPAP, systolic pulmonary artery pressure. Elevated RV/LV ratio was defined as dilated RV with basal RV/LV ratio >1.0. sPESI, simplified pulmonary embolism severity index. *The difference is statistically significant.
