# Supplementary material for: Clinical Characteristics and Prognosis of Acute Pulmonary Embolism with Hemoptysis in Autoimmune Disease Patients
Source: Int J Med Sci. 2024 May 19;21(8):1399–407. doi: 10.7150/ijms.94052 (PMC11186428; doi:10.7150/ijms.94052)
Supplement: Supplementary file 1 — Supplementary tables. [file ijmsv21p1399s1.pdf]

Supplementary material

Table S1. Clinical characteristics of PE patients with hemoptysis versus patients without hemoptysis

	Hemoptysis group (n=105/896,11.7%)	Non-hemoptysis group (n=791/896,88.3%)	***P*-value**
**Male, n (%)**	64 (61.0)	329 (41.6)	<0.001*
**Age, year, mean ± SD**	48.9±18.5	59.8±15.8	<0.001*
**Age < 48 years old, n (%)**	54 (51.4)	162 (20.5)	<0.001*
**BMI, kg/m^2, mean ± SD**	24.85±4.41	24.64±3.47	0.262
**Symptoms, n (%)**			
Chest pain	44 (41.9)	79 (10.0)	<0.001*
Dyspnea	58 (55.2)	494 (62.5)	0.106
Syncope	5 (4.8)	85 (10.7)	0.109
**Signs, n (%)**			
Temperature< 36◦C	1 (1.0)	17 (2.1)	0.712
Pulse ≥ 110 beats/min	16 (15.2)	172 (21.7)	0.175
RR > 30 breaths/min	13 (12.4)	36 (4.6)	0.001*
SBP < 100 mmHg	13 (12.4)	107 (13.5)	0.712
SpO_2＜90%	26 (24.8)	232 (29.3)	0.325
**Comorbid Diseases, n (%)**			
Coronary atherosclerotic heart disease	10 (9.5)	87 (11.0)	0.648
Chronic Heart failure	17 (16.2)	57 (7.2)	0.002*
COPD/Asthma	5 (4.8)	38 (4.8)	>0.99
Pulmonary infection	31 (29.5)	138 (17.4)	0.003*
DPLD	2 (1.9)	45 (5.7)	0.157
Bronchiectasis	0	4 (0.5)	>0.99
Primary/Metastatic lung cancer	22 (21.0)	82 (10.4)	0.001*
Diabetes mellitus	10 (9.5)	125 (15.8)	0.109
Autoimmune disease	41 (39.0)	127 (16.1)	<0.001*
SLE/SS/RA/UCTD/IIM	16 (15.2)	76 (9.6)	0.136
APS	14 (13.3)	44 (5.6)	0.001*
Vasculitis	16 (15.2)	26 (3.3)	<0.001*
**Laboratory findings**			
WBC (× 10^9/L), median (IQR)	8.41 (5.79,12.28)	8.75 (6.40,12.01)	0.532
Anemia (HGB<90g/L), n (%)	5 (4.8)	120 (15.2)	0.004*
PLT (× 10^9/L), median (IQR)	191 (134, 275)	196 (146, 264)	0.365

PLT < 80 × 10^9/L, n (%)	16 (15.2)	53 (6.7)	0.004*
Hypoalbuminemia, n (%)	23 (21.9)	190 (24.0)	0.783
D-dimer (mg/L), median (IQR)	4.31 (2.07,11.81)	5.00 (2.26,11.18)	0.506
cTnI ≥ 0.056ug/L, n (%)	22 (21.0)	239 (30.2)	0.135
NT-proBNP (pg/ml), median (IQR)	180 (125,1285)	478 (136,1878)	0.002*
NT-proBNP > 216 pg/ml, n (%)	33 (31.4)	420 (53.1)	<0.001*
Cr (umol/L), median (IQR)	70 (58,84)	66 (55,84)	0.100
**Echocardiography, n (%)**			
Elevated RV/LV ratio	12 (11.4)	132 (16.7)	0.041*
RV free wall hypokinesis	8 (7.6)	53 (6.7)	0.818
sPAP≥50mmHg	14 (13.3)	96 (12.1)	0.309
**CTPA, n (%)**			
Central emboli	11 (10.5)	96 (12.1)	0.760
**sPESI, n (%)**			
0	40 (38.1)	179 (22.6)	0.002*
1	32 (30.5)	283 (35.8)	0.337
2	20 (19.0)	233 (29.5)	0.035*
3	11 (10.5)	75 (9.5)	0.647
4	2 (1.9)	18 (2.3)	0.834

*SD, standard deviation; BMI, body mass index; RR, respiratory rate; SBP, systolic blood pressure; COPD, chronic obstructive pulmonary disease; DPLD, diffuse parenchymal lung disease; SLE, Systemic Lupus Erythematosus; SS, Sjögren's syndrome; RA, Rheumatoid Arthritis; UCTD, Undifferentiated connective tissue disease; IIM, idiopathic inflammatory myopathy; APS, antiphospholipid syndrome; WBC, white blood cell; IQR, interquartile range; HGB, hemoglobin; PLT, platelet; cTnI, cardiac troponin I; NT-proBNP, N-terminal pro-B-type natriuretic peptide; RV, right ventricle; LV, left ventricle; sPAP, systolic pulmonary artery pressure; sPESI, simplified pulmonary embolism severity index. Elevated RV/LV ratio was defined as dilated RV with basal RV/LV ratio >1.0.*The difference is statistically significant.*

Table S2. Univariate analysis and multivariate analysis of clinical factors associated with hemoptysis in PE patients.

**Characteristic**	**Univariate Analysis**			**Multivariate Analysis**		
	**OR**	**95% CI**	**P value**	**OR**	**95% CI**	**P value**
Male	2.28	1.51,3.49	<0.001*	1.93	1.21,3.1	0.006*
Age < 48 years old	4.11	2.70, 6.27	<0.001*	4.22	2.51,7.09	<0.001*
Chest pain	6.25	3.96, 9.83	<0.001*	5.09	3.08,8.44	<0.001*
Dyspnea	0.71	0.47, 1.08	0.107	-	-	-
Syncope	0.50	0.19, 1.09	0.115	-	-	-
Coronary atherosclerotic heart disease	0.85	0.40, 1.62	0.648	-	-	-
Chronic Heart failure	2.49	1.35, 4.38	0.002*	3.33	1.68,6.63	<0.001*
COPD/Asthma	0.99	0.34, 2.36	0.985	-	-	-
Pulmonary infection	1.98	1.24, 3.11	0.003*	2.47	1.45,4.21	<0.001*
DPLD	0.32	0.05, 1.06	0.121	-	-	-
Primary/Metastatic lung cancer	2.29	1.33, 3.81	0.002*	5.21	2.76,9.84	<0.001*
Diabetes mellitus	0.56	0.27, 1.06	0.095	-	-	-
Autoimmune Disease	3.49	2.25, 5.37	<0.001*	2.62	1.5,4.59	<0.001*
PLT＜80 × 10^9/L	2.50	1.34, 4.47	0.003*	1.26	0.59,2.68	0.549
Central emboli	0.85	0.42, 1.59	0.641	-	-	-

COPD, chronic obstructive pulmonary disease; DPLD, diffuse parenchymal lung disease; PLT, platelet.
**The difference is statistically significant.*

Table S3. Short-term mortality of PE patients with hemoptysis versus patients without hemoptysis

Characteristics	Patients with hemoptysis (n=105)	Patients without hemoptysis(n=791)	P-value
**All-cause deaths, n (%)**			
7-day	3 (2.9)	31 (3.9)	0.788
14-day	7 (6.7)	51 (6.4)	0.835
30-day	12 (11.4)	84 (10.6)	0.801
